# Comparable outcomes of BTK inhibitors and fixed-duration venetoclax plus rituximab in second-line treatment of chronic lymphocytic leukaemia: a real-world analysis by the Czech CLL study group

**DOI:** 10.1007/s00277-026-07043-8

**Published:** 2026-05-13

**Authors:** Jana Mihályová, Anna Panovská, Martin Šimkovič, Lukáš Smolej, Martin Špaček, Tereza Shokralla, Zuzana Kubová, Jana Zuchnická, Tomáš Arpáš, Pavel Vodárek, Peter Turcsányi, Lenka Polcerová, Marika Chrápavá, Daniel Lysák, Martin Brejcha, Heidi Móciková, Michael Doubek

**Affiliations:** 1 Department of Hematooncology, University Hospital Ostrava, Ostrava, Czech Republic; 2 University of Ostrava, Ostrava, Czech Republic; 3 Department of Internal Medicine – Hematology and Oncology, University Hospital Brno and Faculty of Medicine, Masaryk University, Brno, Czech Republic; 4 4th Department of Internal Medicine–Hematology, Faculty of Medicine in Hradec Králové, University Hospital and Charles University in Prague, Hradec Králové, Czech Republic; 5 1st Department of Medicine - Haematology, First Faculty of Medicine, Charles University and General University Hospital in Prague, Prague, Czech Republic; 6 Department of Hemato-Oncology, Faculty of Medicine and Dentistry, Palacky University and University Hospital, Olomouc, Czech Republic; 7 Institute of Biostatistics and Analyses, Ltd, Brno, Czech Republic; 8 Department of Hematology and Oncology, Medical School and Teaching Hospital in Plzen, Charles University in Prague, Plzen, Czech Republic; 9 Hospital Agel, Nový Jičín, Czech Republic; 10 Department of Hematology, Fakultni nemocnice Kralovske Vinohrady and 3rd Faculty of Medicine, Charles University, Prague, Czech Republic; 11 Center of Molecular Medicine, Central European Institute of Technology, Masaryk University, Brno, Czech Republic

**Keywords:** Ibrutinib, Acalabrutinib, Venetoclax, Rituximab, Chronic lymphocytic leukaemia

## Abstract

In relapsed/refractory (RR) chronic lymphocytic leukaemia (CLL), Bruton tyrosine kinase inhibitors (BTKi) are administered as monotherapy until disease progression. In contrast, venetoclax, a B-cell lymphoma 2 (BCL-2) protein inhibitor, is typically combined with rituximab (VenR) as a time-limited regimen. To date, neither randomized nor retrospective trials have directly compared these approaches in RR CLL, and only limited data are available comparing venetoclax plus obinutuzumab (VenObi) to BTKi in the first-line setting. We retrospectively analysed 352 patients receiving second-line therapy: 93 VenR and 259 BTKi (ibrutinib: *n* = 222; acalabrutinib: *n* = 37). Baseline characteristics were well balanced, except for a higher incidence of *TP53* mutation and trisomy 12 in BTKi-treated patients. At a median follow-up of 13.8 months (VenR) and 22.1 months (BTKi), 12-month progression-free survival (PFS) and overall survival (OS) were comparable (PFS: 85.1% vs. 82.2%, *p* = 0.265; OS: 87.6% vs. 88.4%, *p* = 0.291). Estimated median PFS (45.4 vs. 37.9 months, *p* = 0.265) and OS (83.6 vs. 74.2 months, *p* = 0.291) also showed no significant differences between the VenR and BTKi cohorts. Treatment discontinuation due to adverse events before month 24 was more frequent with BTKi (22.5% with VenR vs. 34.3% with BTKi), primarily due to infections (10.6% vs. 28.6%) and disease progression (14.3% vs. 22.5%). These preliminary data suggest comparable outcomes and manageable toxicity profiles for both regimens.

**Supplementary Information:**

The online version contains supplementary material available at 10.1007/s00277-026-07043-8.

## Introduction

Chronic lymphocytic leukaemia (CLL) is characterized by a highly heterogeneous clinical course influenced by several cytogenetic and molecular biomarkers. Adverse prognostic factors include *TP53* aberrations (deletion 17p [del(17p)] and/or *TP53* mutation), del(11q), and unmutated immunoglobulin heavy chain variable region (*IGHV*). Trisomy 12 is generally considered an intermediate-risk feature, whereas isolated del(13q) is associated with a favourable disease course [1–4]. In the era of targeted therapies, treatment selection is influenced mainly by the presence of *TP53* aberrations, while other biomarkers have a limited impact on therapeutic decision-making [3, 4].

Over the past decade, Bruton tyrosine kinase (BTK) inhibitors (i) and B-cell lymphoma 2 (BCL-2) protein inhibitors have revolutionized the treatment of CLL, leading to substantial improvements in both progression-free (PFS) and overall survival (OS) outcomes [5–10].

Ibrutinib was the first covalent (irreversible) BTK inhibitor approved for CLL treatment [5, 6]. Since its approval, new generations of these drugs have been developed. Currently, acalabrutinib and zanubrutinib represent alternatives to ibrutinib [7, 8] and many others are under investigation (e.g. tirabrutinib, orelabrutinib). Non-covalent (reversible) BTK inhibitors (e.g. pirtobrutinib, nemtabrutinib) have been designed to address some limitations of their predecessors, with pirtobrutinib already offering an effective treatment option after covalent agents [11–13]. Across all registrational clinical trials, covalent BTK inhibitors used as monotherapy in relapsed/refractory (RR) CLL showed long-lasting remissions with median PFS ranging from 44.1 months to not yet reached (depending on the follow-up) and overall response rates (ORR) ranging between 83% and 91% [5–8].

Venetoclax is currently the only BCL-2 inhibitor approved for CLL therapy. Early clinical trials demonstrated its efficacy as monotherapy in patients previously treated with covalent BTK inhibitors. Response rates ranged from 65% to 79.4% [14, 15]. Subsequent studies assessed the fixed-duration combination of venetoclax plus rituximab (VenR) in RR CLL and reported an ORR, complete remission (CR) rate, and median PFS of 92%, 26.8%, and 53.6 months, respectively. Furthermore, 62% of evaluable patients achieved undetectable minimal residual disease (MRD), reflecting the depth of response associated with this regimen [16–18].

In RR CLL, randomized comparisons between BTK inhibitors and VenR are lacking. Available data are limited to two comparative trials of BTK inhibitors: ALPINE (zanubrutinib versus ibrutinib) and ELEVATE-RR (acalabrutinib versus ibrutinib) [7, 19]. In these trials, zanubrutinib demonstrated improved PFS, while both second-generation BTKi agents showed a more favourable cardiovascular safety profile than ibrutinib. The optimal sequencing of these two treatment approaches therefore remains unclear.

In the Czech Republic, VenR along with either ibrutinib, acalabrutinib or zanubrutinib monotherapy represent the most common regimens currently indicated for the treatment of RR CLL. As all these regimens share identical indications in the RR CLL setting, treatment selection in real-world practice depends on the drug toxicity profile, patient comorbidities (e.g. impaired renal function, hypertension, cardiac arrhythmias, indications for long-term anticoagulation), baseline disease characteristics (e.g., presence of *TP53* aberrations, tumour burden, risk of tumour lysis syndrome and platelet count), as well as individual preferences. Czech national guidelines recommend continuous BTK inhibitor therapy for patients with *TP53* aberrations. However, this approach is not strictly mandated, and patients may receive the time-limited VenR regimen.

To evaluate whether BTK inhibitors until progression or fixed-duration venetoclax therapy are more rational and effective, we conducted a retrospective analysis of the second-line treatment. This study aimed to optimize treatment sequencing by providing comprehensive data on the clinical efficacy and safety in the real-world setting.

## Patients and methods

### Study design

This retrospective, multicentre analysis evaluated CLL patients who received second-line therapy with either a fixed-duration VenR regimen or one of the BTK inhibitors, specifically ibrutinib or acalabrutinib. Data analysed were captured from the Czech National Registry of patients with CLL, the Chronic Lymphocytic Leukemia Patients Registry (CLLEAR), run by the Czech CLL Study Group and the Czech Leukaemia Study Group. Consecutive patients enrolled in the registry between December 2015 and February 2024 were included in the final analysis. Patients were treated in one of the eight Czech haematological centres cooperating within the Czech CLL Study Group. Inclusion criteria for treatment commencement, staging and treatment response were assessed according to the guidelines of the International Workshop on Chronic Lymphocytic Leukaemia 2018 criteria (iwCLL) [4]. Cumulative Illness Rating Scale (CIRS) score and National Cancer Institute Common Terminology Criteria for Adverse Events (CTCAE) version 5.0 were used for comorbidity and adverse event assessment [20]. Key prognostic biomarkers were assessed as part of routine diagnostic work-up before treatment initiation in all patients. *IGHV* mutational status was determined using an RT-qPCR–based gene expression assay [21], *TP53* mutations were detected by Sanger sequencing or next-generation sequencing (NGS) [22]. Cytogenetic abnormalities, including del(17p), del(11q), del(13q) and trisomy 12, were assessed by fluorescence in situ hybridization (FISH). Conventional chromosome banding analysis (G-banding) was performed to evaluate the karyotype. Karyotypes were reported according to the International System for Human Cytogenomic Nomenclature (ISCN), with evaluation of at least 20 metaphases when available. The protocol was approved by the independent ethics committees at each site. The study was conducted in compliance with the Declaration of Helsinki and International Committee on Harmonization - Good Clinical Practice guidelines. All patients provided written informed consent before key CLL-related clinical and laboratory parameters were obtained from the registry.

### Patients

Adult CLL patients indicated for second-line treatment who had received a chemoimmunotherapy-based regimen in the first-line setting were included in the study (*N* = 352). Those who received a BTK inhibitor, BCL-2 inhibitor, or phosphatidylinositol 3-kinase delta (PI3Kδ) inhibitor as first-line treatment, were excluded. Patients were divided into two cohorts: individuals who were administered one of BTK inhibitors (ibrutinib, acalabrutinib) and those who were treated with VenR regimen (Table 1). Ibrutinib and acalabrutinib monotherapy were given at standard doses (ibrutinib: 420 mg orally once a day, acalabrutinib: 100 mg orally every 12 h) until disease progression (PD) or unacceptable toxicity. The combination of VenR was administered in the regular dosing schedule for 24 months (6 cycles of rituximab intravenously or as a subcutaneous injection and 24 cycles of venetoclax, starting from 20 mg orally once a day and increasing to the target dose of 400 mg over 5 weeks).

**Table 1:** Patient characteristics

	VenR		BTKi		*p*-value
	*N* = 93		*N* = 259		
Male gender, n (%)	60 (64.5%)		164 (63.3%)		0.837
Median age, years (range)	70.0 (45–86)		71.3 (34–90)		0.264
Median time from dg to the 2nd line treatment, months (range)	59.9 (4–324)		58.2 (2–379)		0.240
Median CIRS score (range)	7.0 (0–15)		7.0 (0–18)		0.757
Clinical stage (Rai), n (%)	*N* = 91		*N* = 239		0.352
0-I	25 (27.5%)		52 (21.8%)		
II	18 (19.8%)		69 (28.9%)		
III	17 (18.7%)		43 (18.0%)		
IV	31 (34.1%)		75 (31.4%)		
Clinical stage (Binet), n (%)	*N* = 90		*N* = 243		0.274
A	17 (18.9%)		32 (13.2%)		
B	33 (36.7%)		109 (44.9%)		
C	40 (44.4%)		102 (42.0%)		
ECOG, n (%)	N = 89		N = 215		0.388
0	24 (27.0%)		68 (31.6%)		
1	56 (62.9%)		134 (62.3%)		
2	9 (10.1%)		13 (6.0%)		
Maximum size of LN, n (%)	N = 90		N = 225		0.364
<2 cm	8 (8.9%)		20 (8.9%)		
2–5 cm	37 (41.1%)		101 (44.9%)		
5–10 cm	23 (25.6%)		68 (30.2%)		
>10 cm	22 (24.4%)		36 (16.0%)		
Absolute lymphocyte count (x10e9/l), n	N = 91		N = 241		0.746
Median (range)	23.5 (0–324)		35.7 (0–455)		
Splenomegaly, n (%)	N = 92		N = 242		0.102
Yes	46 (50.0%)		145 (59.9%)		
Tumour lysis syndrome risk (TLS), n (%)	N = 36		N = 78		0.944
Low	19 (52.8%)		40 (51.3%)		
Intermediate	10 (27.8%)		24 (30.8%)		
High	7 (19.4%)		14 (17.9%)		
Unknown	57		181		
Creatinine clearance, ml/min, median (range)	67 (35.6–52.6)		64.7 (34.0-69.2)		0.325
IGVH mutation, n (%)	N = 93		N = 259		0.819
Unmutated	82 (88.2%)		226 (87.3%)		
TP53 mutation and/or deletion 17p, n (%)	N = 93		N = 259		0.244
Yes	25 (26.8%)		97 (37.4%)		
TP53 mutation, n (%)	N = 93		N = 259		0.010
Yes	15 (16.1%)		77 (29.7%)		
Deletion 17p, n (%)	N = 93		N = 259		0.791
Yes	21 (22.6%)		62 (23.9%)		
Deletion 11q, n (%)	N = 89		N = 226		0.574
Yes	29 (32.6%)		86 (38.1%)		
Deletion 13q, n (%)	N = 89		N = 226		0.315
Yes	43 (48.3%)		124 (54.9%)		
Trisomy 12, n (%)	N = 89		N = 226		0.004
Yes	10 (11.2%)		32 (14.2%)		
Complex karyotype†, n (%)	N = 45		N = 130		0.677
Yes	15 (33.3%)		39 (30.0%)		

*N/n* number, *dg* diagnosis, *LN* lymph node, †Complex karyotype, 3 and more chromosomal abnormalities

### Outcomes

The primary endpoint was progression-free survival. Secondary endpoints included overall response rate, complete remission rate, overall survival, time to next treatment (TTNT), and adverse events. The prognostic value of *IGHV* mutation status and *TP53* aberrations (*TP53* mutation and/or deletion in the short arm of chromosome 17) was prespecified and evaluated in relation to treatment response and survival outcomes.

## Statistics

Statistical analyses were conducted using the IBM Statistical Package for the Social Sciences (SPSS, version 29.0) and R software (version 4.2.3). The PFS was defined as the time from treatment initiation to disease progression/relapse or death from any cause. The OS was defined as the time from treatment initiation to death from any cause. The TTNT was defined as the time from treatment initiation to the date of the next treatment line initiation. Treatment discontinuation without documented disease progression was not considered an event. Patients who discontinued treatment early were followed until disease progression (PD), death, or last follow-up. Kaplan–Meier curves were used to estimate PFS, OS and TTNT, and differences between treatment groups were compared using the log-rank test. Median follow-up was calculated using the reverse Kaplan–Meier method. Differences in categorical variables between treatment groups were assessed using Pearson’s chi-squared test or Fisher’s exact test, as appropriate. Continuous variables were compared using the non-parametric Mann–Whitney test. A p value < 0.05 was considered statistically significant; all p values were two-sided.

## Results

In total, 352 patients who entered the CLLEAR registry between December 2015 and February 2024 were selected for the final analysis. Out of those patients, 93 were treated with VenR regimen and 259 were administered one of the BTK inhibitors (ibrutinib: *n* = 222; acalabrutinib: *n* = 37). Baseline patient characteristics and prognostic markers were well balanced (Table 1). The median age at the start of the second-line treatment was 70.0 (range, 45–86) and 71.3 (range, 34–90) years (*p* = 0.180) in the VenR and BTKi cohorts, respectively. Men prevailed in both groups and no statistically significant differences were seen in clinical stage, CIRS score, creatinine clearance, *IGHV* mutational status, and most cytogenetic abnormalities. Among high-risk molecular features, *TP53* mutations (16.1% vs. 29.7%, *p* = 0.010), and trisomy 12 (11.2% vs. 14.2%, *p* = 0.004), were more frequently observed in patients who received BTK inhibitors. The combinations of fludarabine, cyclophosphamide plus rituximab (FCR) and bendamustine plus rituximab (BR) were the most common regimens used in the first-line treatment (ESM 1).

### Efficacy

The median follow-up was longer in the BTKi group: 13.8 months (range, 0–111) in the VenR compared with 22.1 months in BTKi cohort (range, 2–84) (*p* = 0.006) (Table 2). All patients were assessed for survival analysis, including PFS, OS and TTNT, while treatment response rates were available for 69 (out of 93) patients treated with VenR and 218 (out of 259) patients treated with ibrutinib or acalabrutinib. At the data cutoff, 53.8% and 52.1% of patients remained on VenR and BTKi treatment, respectively (Table 2, ESM 2). The ORR was similar between the groups (95.7% vs. 89.9% *p* = 0.218), though complete remissions, including CR with incomplete bone marrow recovery (CRi), were more frequent in the VenR arm (42% vs. 13.3%, *p* < 0.001) (Table 2). Of note, in a subset of patients, CR was assessed based on clinical, laboratory and radiographic criteria without bone marrow confirmation, which may overestimate CR rates in our analysis.

**Fig. 1:**
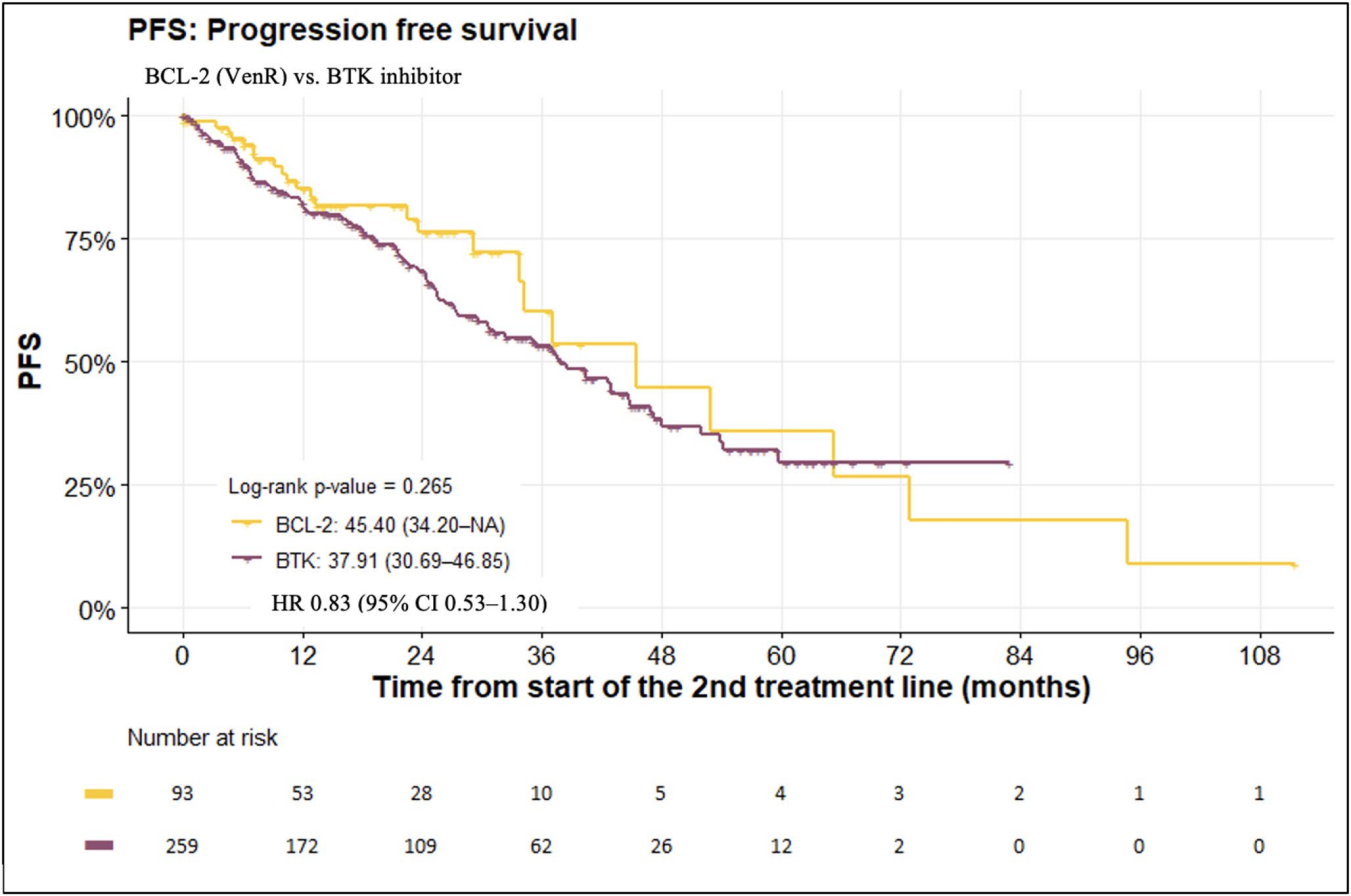
Progression free survival (PFS)

The 12-month PFS was 85.1% (95% confidence interval [CI], 77.2–93.7) with VenR and 82.2% (95% CI, 77.4–87.3) with BTKi **(**HR 0.83, 95% CI 0.53–1.30; *p* = 0.265**)** (Table 3; Fig. 1). A subanalysis of patients with TP53 aberrations showed comparable 12-month PFS between the two arms (78.2% vs. 86.1%; *p* = 0.244), and a similar finding was observed in the unmutated *IGHV* population (83.5% vs. 81.2%; *p* = 0.154) (ESM 3–6).

**Table 2:** Overall response rate

		VenR		BTKi		*p*-value
		N = 69		N = 218		
ORR, n (%)		66 (95.7%)		196 (89.9%)		0.218
CR		26 (37.7%)		27 (12.4%)		< 0.001
CRi		3 (4.3%)		2 (0.9%)		
PR		37 (53.6%)		167 (76.6%)		
SD		0 (0.0%)		10 (4.6%)		
PD		3 (4.3%)		12 (5.5%)		
MRD Peripheral blood, n (%)		*N* = 31		*N* = 24		< 0.001
Positive		14 (45.2%)		23 (95.8%)		
Negative		17 (54.8%)		1 (4.2%)		
		*N* = 93		*N* = 259		
Median Duration of treatment, months (range)		10.4 (0–28)		12.4 (0–59)		0.697
Median Follow-up, months (range)		13.8 (0–111)		22.1 (2–84)		0.006

The best achieved response is reported *N/n* number, *CR* complete remission,* CRi* complete remission with incomplete bone marrow recovery, *PR* partial remission, *SD* stable disease,* PD* progression disease, *MRD* minimal residual disease, *ORR *overall response rate

During the follow-up period, death was reported in 19 (20.4%) and 62 (23.9%) patients in the VenR and BTKi groups, respectively. The main causes of mortality were infections (10.5% vs. 41.9%), CLL progression (26.3% vs. 22.6%), and secondary malignancies (21.1% vs. 14.5%) (ESM 7–8). A substantial proportion of deaths occurred during 2020–2022 (VenR: 21.5%, BTKi: 20.1% patients), reflecting the impact of the COVID-19 pandemic. A cardiovascular cause of death was reported in 2 patients treated with BTK inhibitor and 1 with VenR regimen (ESM 7). Information on whether the death was drug-related was not provided.

**Fig. 2:**
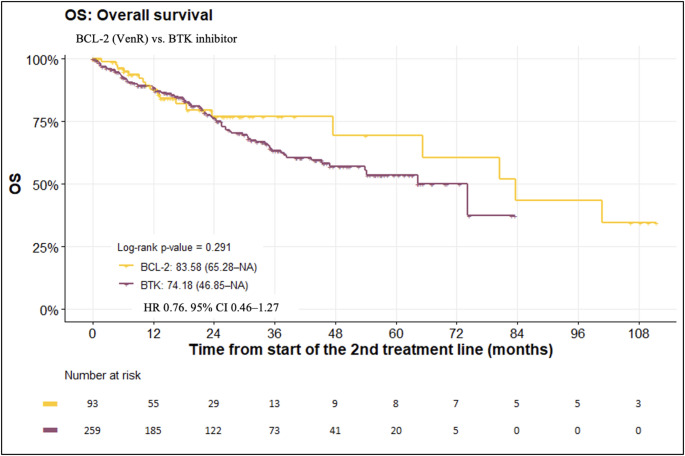
Overall survival (OS)

The 12-month OS was comparable between the two treatment regimens, at 87.6% (95% CI, 80.3–95.6) with VenR and 88.4% (95% CI, 84.4–92.6) with BTKi **(**HR 0.76, 95% CI 0.46–1.27; *p* = 0.291**)** (Table 3; Fig. 2). A subgroup analysis based on either unmutated *IGHV*, or *TP53* mutations showed no statistically significant difference in 12-month OS between the treatment groups (ESM 9–12).

**Table 3:** Progression-free survival & overall survival

	VenR		BTKi		*p*-value
	*N* = 93		*N* = 259		0.265
Progression-free survival					
n events (%)	24 (25.8%)		107 (41.3%)		
KM median (95% CI)	45.4 (34.2–NA)		37.9 (30.7–46.9)		
Probability at time, % (95% CI):					
12 months	85.1 (77.2–93.7)		82.2 (77.4–87.3)		
24 months	76.3 (65.9–88.3)		68.3 (62.1–75.1)		
36 months	60.2 (44.1–82.2)		53.3 (46.2–61.4)		
48 months	44.6 (26.3–75.6)		38.2 (30.4–48.0)		
60 months	35.7 (18.0–70.8)		29.5 (21.2–40.9)		
Overall survival	VenR		BTKi		p-value
	*N* = 93		*N* = 259		0.291
n events (%)	19 (20.4%)		77 (29.7%)		
KM median (95% CI)	83.6 (65.3–NA)		74.2 (46.9–NA)		
Probability at time, % (95% CI):					
12 months	87.6 (80.3–95.6)		88.4 (84.4–92.6)		
24 months	77.0 (66.6–89.0)		76.2 (70.5–82.4)		
36 months	77.0 (66.6–89.0)		63.5 (56.5–71.3)		
48 months	69.3 (53.8–89.2)		57.0 (49.3–65.9)		
60 months	69.3 (53.8–89.2)		53.6 (45.3–63.4)		

Abbreviation: N: number

Time to next treatment was evaluated in 85 patients treated with VenR and 251 patients treated with BTKi. There was no significant difference in TTNT between the treatment groups, with an estimated median of 45.4 months (95% CI, 35.3–NA) in the VenR group and 53.4 months (95% CI, 44.8–NA) in the BTKi group **(**HR 0.70, 95% CI 0.38–1.29; *p* = 0.764**).** Among BTKi patients, the estimated median TTNT was shorter in cases with TP53 aberrations compared with those without (44.8 vs. 60.3 months, *p* = 0.041), whereas this difference was not observed in the VenR group (ESM 13–18).

### Safety

Hematologic toxicity of any grade was reported in 58.1% and 32.0% of patients in the VenR and BTKi regimens, respectively. Neutropenia, the most common grade 3–4 hematologic toxicity, occurred more frequently in the VenR arm: VenR 35.4% vs. BTKi 10.1%. Frequency of severe (grade ≥3) anaemia (6.5% vs. 1.9%) and thrombocytopenia (10.7% vs. 3.8%) was comparable. Non-hematologic adverse events (AEs) of any grade were recorded in 18.3% and 48.7% of patients in the VenR and BTKi arm. Infections grade ≥ 3 developed in 7.5% and 14.3%, respectively (ESM 19). Hypertension of any grade associated with BTKi were reported in 12% of patients and no tumour lysis syndrome (TLS) was reported in the VenR group (ESM 20). Treatment modifications due to any cause were more frequent among BTKi patients: VenR 17.2% vs. BTKi 35.9% (*p* = 0.001). Additionally, more individuals in the BTKi cohort discontinued treatment before month 24 (34.7% vs. 22.1%, *p* < 0.001), with an additional 11.9% discontinuing BTKi treatment thereafter (ESM 2).

## Discussion

The European Society for Medical Oncology (ESMO) updated guidelines for CLL treatment in 2024. Patients who relapse after chemoimmunotherapy are recommended to receive either a BTK inhibitor as continuous therapy (acalabrutinib and zanubrutinib are preferred over ibrutinib) or a time-limited VenR regimen. Rituximab plus idelalisib or venetoclax monotherapy may be considered under certain conditions, especially in cases with TP53 mutations or del(17p) [3]. The Czech national treatment guidelines are in line with the ESMO recommendations (https://cll.cz/cs/obsah/doporuceni).

Given the overlapping indications of BTK inhibitors and VenR in RR CLL, their distinct drawbacks highlight the need for individualized treatment selection and further investigation into optimal sequencing and combination strategies. Therapy with BTK inhibitors is primarily disease controlling rather than curative, with low rates of CR. Long-term use is associated with a substantial risk of acquired resistance (28%) most commonly driven by *BTK*^*C48*1^ and/or *PLCG2* mutations [23]. Additionally, treatment-related toxicity, particularly cardiovascular events (hypertension, atrial fibrillation/flutter) and bleeding, is a notable concern with ibrutinib, leading to treatment discontinuation in approximately half of patients over five years [7, 19]. Although new generations of BTK inhibitors (e.g. acalabrutinib, zanabrutinib) offer improved tolerability, they share similar resistance mechanisms [24, 25]. On the other hand, VenR, which is associated with deep remissions, requires careful initiation due to the risk of TLS and ongoing management of cytopenia (neutropenia and thrombocytopenia). Resistance is less common in the fixed-duration setting; however, mechanisms such as BCL-2 Gly101Val mutations and upregulation of alternative anti-apoptotic proteins (e.g., MCL1, BCL-XL) have been described [26, 27].

Head-to-head comparisons of these drugs in RR CLL have been confined to a few randomized trials of BTK inhibitors [7, 19], with no direct comparisons available between the VenR and BTKi agents. Herein, we provide real-world data from a direct comparative analysis of the VenR and BTK inhibitors (ibrutinib and acalabrutinib) in the first relapse after chemoimmunotherapy. A disproportionate number of patients and differing follow-up between the two groups reflect the timing of national reimbursement for each treatment. Because only a minority of patients received acalabrutinib, ibrutinib and acalabrutinib were assessed as a single cohort. Zanubrutinib received regulatory approval in the Czech Republic after the conclusion of the study period and, therefore, was not included in this analysis. Baseline patient characteristics were well balanced between the two groups, except for *TP53* mutations and trisomy 12, which were more frequent in the BTKi cohort. The overrepresentation of *TP53* mutations in the BTKi arm is consistent with both international and Czech national guidelines, which recommend BTKi therapy for patients harbouring these high-risk molecular features.

In time-to-endpoint analysis, 12-month PFS and OS rates were similar between VenR and BTKi regimens. The differences in estimated median PFS (45.4 vs. 37.9 months) and OS (83.6 vs. 74.2 months) did not reach statistical significance. These results should be interpreted with caution as longer follow-up is needed to confirm our predictions (Table 3; Figs. 1 and 2). The 12-month PFS in our BTKi cohort is comparable to outcomes reported in RESONATE and ASCEND trial [5, 28]. However, the median PFS of 44.1 months in the RESONATE study [6] exceeds that of our BTKi group. Similarly, while the 12-month PFS in our VenR cohort corresponds with MURANO trial, the median PFS is shorter than the reported 53.6 months [16, 29]. Cross-trial subanalyses suggest a long-term benefit of continuous BTKi therapy in patients with *TP53* aberration, though the observation has not yet been confirmed in our analysis (ESM 4, 6, 15, 18). The differences may reflect the small sample size and relatively short follow-up, which may not capture the long-term benefits of continuous BTKi treatment.

Despite discrepancies with registrational studies, our results are comparable with real-world data published to date. For instance, the PFS observed in the BTKi cohort is consistent with our previous analysis comparing ibrutinib to rituximab plus idelalisib (median PFS for ibrutinib: 40.5 months) [30], as well as with the median PFS (35 months) reported in a large U.S. retrospective trial of ibrutinib [31]. Real-world evidence (RWE) on acalabrutinib efficacy remains limited [32]. The Czech RWE research comparing ibrutinib and acalabrutinib in RR CLL showed median TTNT of 51.6 and 60.1 months, respectively (Arpáš et al., iwCLL 2025), which corresponds with the ibrutinib TTNT observed in the present analysis (ESM 13–18).

Clinical data on venetoclax treatment in RR CLL has emerged from multiple international cohorts. Italian studies reported 12-month PFS rates of 87.2% for VenR [33] and 82% for both VenR and Ven [34]. The Polish Adult Leukemia Group documented a median PFS of 36.97 months for VenR [35] and the International Collaborative Study of Real-World Evidence (CORE) reported a median PFS of 43 months in high-risk population (unmutated *IGHV* or TP53 aberrations) [36]. Additionally, a separate CORE analysis of patients previously treated with BTK inhibitors, confirmed median PFS of 43.2 months [37] (Table 4).

**Table 4:** Selected real-world studies

	Ibrutinib	Ibrutinib	VenR	Ven+VenR	Ven+VenR	VenR	VenR
Publication	Spacek et al., 2023	Mato et al., 2018	Ysebaert et al., 2022	Scarfo et al., 2021	Coombs et al., 2024	Gosh et al., 2024	Sobon et al., 2023
Number of patients	244	536$	70	124	78	60	117
Median age, years	69	60	73	70	65	68	67
Male Gender, %	70	NA	67	66	NA	69	62
Median previous line of therapy, n	2	≥ 1	2	2	1	1–2	2
Unmutated IGVH, %	88	NA	NA	77%	63	70	NA
TP53 aberration, %	44	13–26	42.4	32	43	17	25
ORR, %	88.5	NA	94	85	85	71	86
CR, %	NA	NA	NA	40	NA	NA	17
Median Follow-up, months	33.7	17	24	14	20	17	20
Median PFS, months	40.5	35	87.2%, 12-month	82%, 12-month	43	86.2%, 12-month	37
Median OS, months	54.4	NR	93%, 24-month	83%, 12-month	NA	NA	NR
Grade 3 neutropenia, %	18	NA	NA	63	NA	NA	NA
Grade 3 infections, %	25	NA	NA	NA	NA	NA	NA
Treatment discontinuation	51%	41%	NA	NA	NA	NA	NA

*ORR* overall response rate, *CR* complete remission, *PFS* progression free survival, *OS *overall survival

Direct comparisons between BTK inhibitors and VenR were presented by Eyre et al. who reported longer PFS in RR CLL patients treated with venetoclax (Ven: *n* = 38; VenR, *n* = 10; ibrutinib: 385), though no OS benefit was observed [38]. A larger group of newly diagnosed CLL patients compared venetoclax plus obinutuzumab (VenObi) to BTK inhibitors. There was a trend towards higher 18-month PFS for VenObi but the difference did not reach statistical significance [39]. Nevertheless, different treatment lines make even indirect comparisons with our patient population challenging.

Regarding efficacy, our data indicate that ORR is comparable between the two cohorts, with a higher CR rate observed in the VenR. This is consistent with findings from randomised clinical trials and RWE [28–31, 37, 38, 40]. Venetoclax-based combinations can achieve deep remissions with undetectable MRD (uMRD), even in the relapsed/refractory setting [18, 41]. In our study, over half of the 31 MRD-evaluated patients on VenR achieved uMRD, aligning with findings from MURANO trial (Table 2). Despite this fact, definitive conclusions should be drawn after a larger proportion of patients reach the MRD assessment time point.

In terms of toxicity, we did not reveal any new safety concerns associated with either VenR or BTKi regimen. No severe bleeding or atrial fibrillation were reported in the BTKi cohort and no TLS was reported in VenR group which likely attributed to underreporting of AEs. During the first two years more patients terminated treatment in the BTKi group. The most common AEs leading to treatment discontinuation in BTKi cohort were: infections (10.8%), disease progressions (22.5%) and deaths (23.3%) which was similar in VenR cohort: infections (28.6%), disease progressions (14.3%) and deaths (14.3%). Three patients who discontinued BTKi therapy underwent allogeneic transplantation (ESM 2 Table S2). The frequency of treatment termination in our study was close to those reported in other real world analyses (BTKi: 22.5%-41%, venetoclax: 17–43%) but higher than those in RESONATE, ASCEND and MURANO trials [16, 18, 33, 34].

The main strengths of our study lie in its well-defined cohort of consecutive, unselected patients with RR CLL from general practice. Unlike most real-world studies, we did not combine VenR with other venetoclax-based regimens, thereby enhancing the analytical power to discern differences between continuous and time-limited therapies. The study’s limitations include unequal patient numbers, relatively short follow-up, potential selection bias, underreporting of AEs, and some statistical constraints. Longer follow-up in the BTKi cohort than in the VenR group may have introduced bias in time-to-event analyses, particularly for TTNT. In addition, multivariable analyses incorporating TP53 mutations and trisomy 12, which were more frequent in the BTKi cohort, could strengthen the interpretation of the observed outcomes.

## Conclusion

Our preliminary analysis suggests that VenR and BTK inhibitors provide comparable efficacy in second-line treatment. Selection of the certain regimen should be guided by factors such as treatment duration, route of drug administration, patient preferences, comorbidities, and toxicity profiles.

## Supplementary information

Below is the link to the electronic supplementary material.

Supplementary Material 1

## Data Availability

All data supporting the findings of this study are available within the paper and its Supplementary Information.
