# Supplementary material for: Comparable outcomes of BTK inhibitors and fixed-duration venetoclax plus rituximab in second-line treatment of chronic lymphocytic leukaemia: a real-world analysis by the Czech CLL study group
Source: Ann Hematol. 2026 May 13;105(8):326. doi: 10.1007/s00277-026-07043-8 (PMC13357439; doi:10.1007/s00277-026-07043-8)
Supplement: Supplementary file 1 — Supplementary Material 1 [file 277_2026_7043_MOESM1_ESM.pdf]

Electronic Supplementary Materials (ESM)

ESM 1: 1st Line treatment		
	**VenR**	**BTKi**
	**N = 93**	**N = 259**
BR, n (%)	20 (21.5%)	99 (38.2%)
FCR, n (%)	48 (51.6%)	90 (34.7%)
R - chlorambucil, n (%)	16 (20.4%)	47 (18.1%)
RCD, n (%)	9 (9.6%)	23 (0.9%)

Abbreviation: N/n: number, B: bendamustine plus rituximab, R: rituximab, FCR: fludarabine, cyclophosphamide plus rituximab, RCD: rituximab, cyclophosphamide, dexamethasone

ESM 2: Treatment modification			
	**VenR**	**BTKi**	**p-value**
	**N = 93**	**N = 259**	
**Dose reduction, n (%)**			**0.001**
No	71 (76.3%)	158 (61.0%)	
Yes	16 (17.2%)	93 (35.9%)	
Unknown	6 (6.4%)	8 (30.8%)	
**Treatment termination, n (%)**			**<0.001**
On schedule†	22 (23.9%)	3 (1.2%)	
Not terminated	50 (53.8%)	135 (52.1%)	
Early‡	21 (22.8%)	121 (46.9%)	
at month 12 or earlier, n (%)	15 (16.1%)	55 (21.2%)	
after month 24, n (%)	0 (0%)	31 (11.9%)	
between month 13 and 24	6 (6.4%)	34 (13.1%)	
**Reason of early treatment termination, n (%)**	**21**	**121**	**0.013**
Death	3 (14.3%)	28 (23.3%)	
Disease progression§	3 (14.3%)	27 (22.5%)	
Inadequate response to therapy	0 (0.0%)	3 (2.5%)	
Haematological toxicity	2 (9.5%)	2 (1.7%)	
Infectious complications	6 (28.6%)	13 (10.8%)	
Comorbidities	1 (4.8%)	5 (4.2%)	
Other	6 (28.6%)	43 (35.5%)	

Abbreviation: N/n: number, ‡VenR: before 24 cycles; BTKi: treatment termination from reason other than PD, §VenR: 3 Richter's Transformations; BTKi: 4 Richter's Transformations, † BTKi: 3 patients indicated for allogeneic transplantation

ESM 3: Progression-free survival according to IGHV mutation status					
	**VenR**	**VenR**	**BTKi**	**BTKi**	**p-value**
	**Unmutated IGHV**	**Mutated IGHV**	**Unmutated IGHV**	**Mutated IGHV**	
	**N = 82**	**N = 11**	**N = 226**	**N = 33**	0.154
n events (%)	24 (29.3%)	0 (0.0%)	97 (42.9%)	10 (30.3%)	
KM median (95% CI)	37.0 (33.8–NA)	–	38.4 (30.6–46.9)	36.9 (25.5–NA)	
Probability at time, % (95% CI):					
12 months	83.5 (75.0–93.0)	100.0 (100.0-100.0)	81.2 (76.0–86.8)	89.2 (78.3–100.0)	
24 months	73.7 (62.4–87.0)	100.0 (100.0-100.0)	68.2 (61.7–75.4)	67.8 (50.1–91.7)	
36 months	56.7 (40.1–80.1)	100.0 (100.0-100.0)	53.2 (45.9–61.8)	52.7 (33.2–83.7)	
48 months	39.7 (21.4–73.6)	100.0 (100.0-100.0)	37.1 (28.9–47.7)	45.2 (26.0–78.5)	
60 months	29.8 (12.9–68.8)	100.0 (100.0-100.0)	27.6 (19.1–39.8)	45.2 (26.0–78.5)	

Abbreviation: del: deletion, w/o: without, N: number

ESM 4: Progression-free survival according to del17p/ mutation TP53

	VenR	VenR	BTKi	BTKi	p-value
	**w/o del17p/TP53**	**del17p/TP53**	**w/o del17p/TP53**	**del17p/TP53**	
	**N = 68**	**N = 25**	**N = 162**	**N = 97**	0.244
n events (%)	10 (14.7%)	14 (56.0%)	66 (40.7%)	41 (42.3%)	
KM median (95% CI)	–	37.0 (29.2–NA)	37.2 (27.3–52.0)	40.4 (30.7–54.2)	
Probability at time, % (95% CI):					
12 months	88.1 (79.5–97.6)	78.2 (62.9–97.3)	79.8 (73.5–86.7)	86.1 (79.1–93.8)	
24 months	78.2 (65.6–93.3)	72.2 (55.2–94.5)	63.4 (55.3–72.6)	76.1 (67.1–86.3)	
36 months	62.6 (39.0–100.0)	55.7 (35.4–87.7)	51.6 (42.8–62.3)	56.2 (45.4–69.6)	
48 months	62.6 (39.0–100.0)	37.1 (18.0–76.7)	38.7 (28.6–52.5)	38.9 (27.8–54.4)	
60 months	62.6 (39.0–100.0)	27.9 (11.1–69.9)	29.1 (18.5–45.5)	32.4 (21.3–49.3)	

Abbreviation: del: deletion, w/o: without, N: number

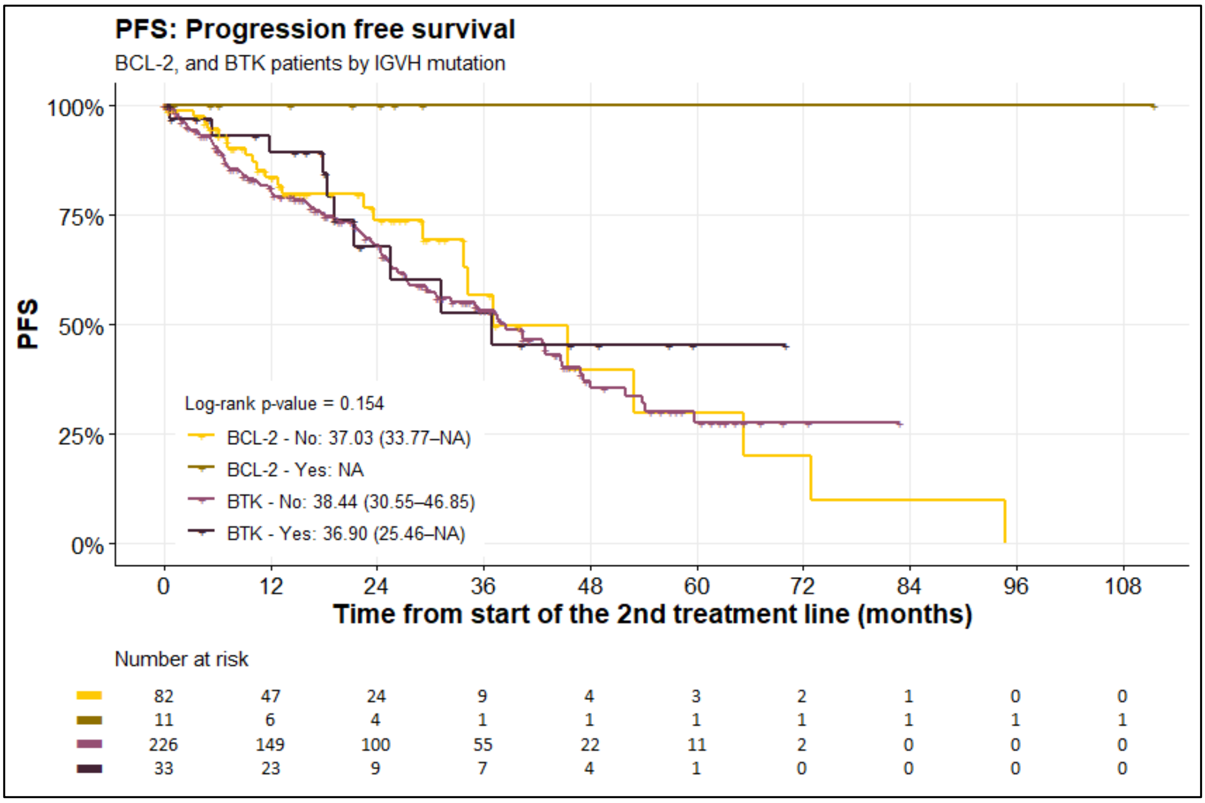

ESM 5: Progression-free survival according to IGHV mutation status

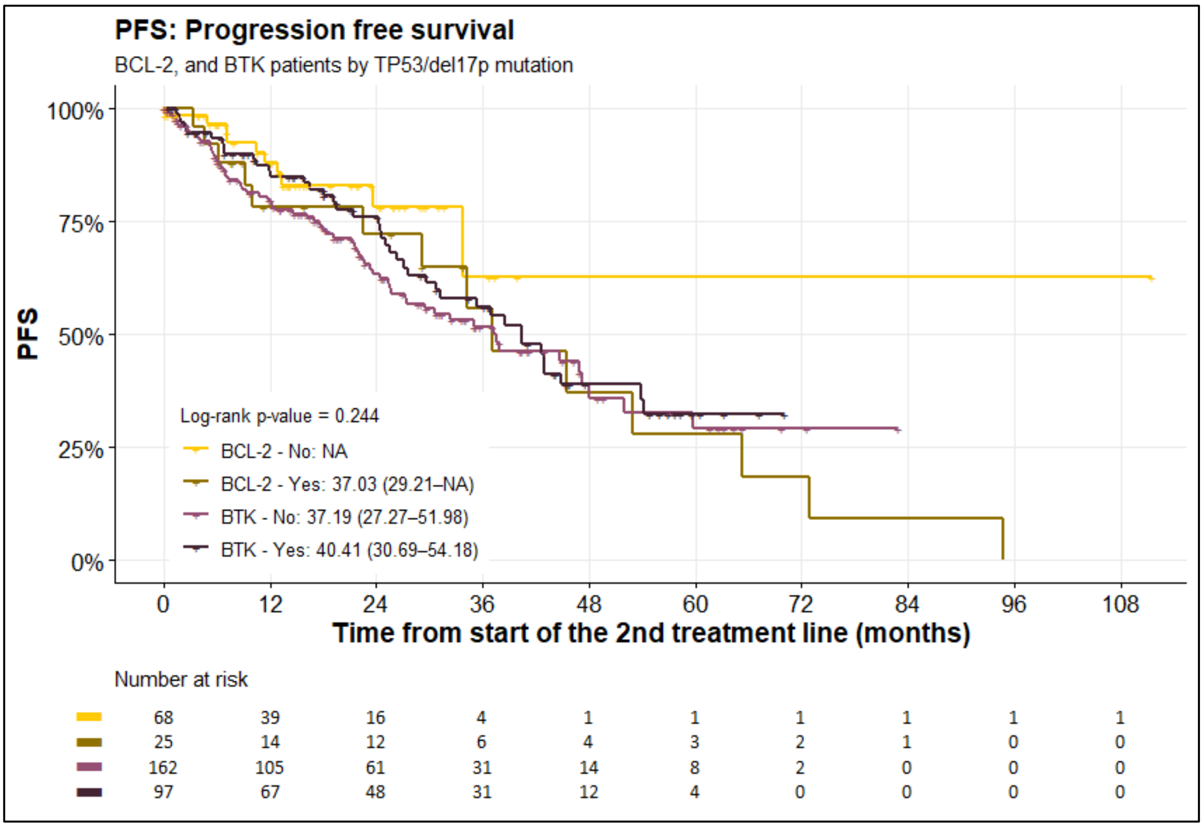

ESM 6: Progression-free survival according to del17p/ mutation TP53

ESM 7: Death within the follow-up period			
	**VenR**	**BTKi**	**p-value**
	**N = 93**	**N = 259**	
**Cause of death, n (%)**	19 (20.4%)	62(23.9%)	0.500
CLL - progression	5 (26.3%)	14 (22.6%)	
CLL - treatment complications	0 (0.0%)	5 (8.1%)	
Another cause	1 (5.3%)	6 (9.7%)	
Infection	2 (10.5%)	26 (41.9%)	
Cardiovascular causes	1 (5.3%)	2 (3.2%)	
Secondary malignancy	4 (21.1%)	9 (14.5%)	

Abbreviation: N/n: number

ESM 8: Secondary primary malignancies			
	**VenR**	**BTKi**	**p-value**
	**N = 3**	**N = 23**	0.938
Pancreatic cancer, n (%)	1 (33.3%)	1 (4.3%)	
Lung cancer, n (%)	1 (33.3%)	3 (13%)	
Non melanoma skin cancer, n (%)	0 (0.0%)	6 (26.1%)	
Kidney cancer, n (%)	0 (0.0%)	2 (8.7%)	
Stomach cancer, n (%)	0 (0.0%)	1 (4.3%)	
Colon cancer, n (%)	0 (0.0%)	1 (4.3%)	
Pancreatic cancer, n (%)	1 (33.3%)	1 (4.3%)	
Melanoma, n (%)	0 (0.0%)	1 (4.3%)	
Prostate cancer, n (%)	0 (0.0%)	1 (4.3%)	
Bladder cancer, n (%)	0 (0.0%)	2 (8.6%)	
Brain cancer, n (%)	0 (0.0%)	1 (4.3%)	
Liver cancer, n (%)	0 (0.0%)	1 (4.3%)	
Bone cancer, n (%)	0 (0.0%)	1 (4.3%)	
Hodgkin lymphoma, n (%)	0 (0.0%)	1 (4.3%)	

Abbreviation: N/n: number

ESM 9: Overall survival according to IGHV mutation status					
	**VenR**	**VenR**	**BTKi**	**BTKi**	**p-value**
	**Unmutated IGHV**	**Mutated IGHV**	**Unmutated IGHV**	**Mutated IGHV**	
n valid	**N = 82**	**N = 11**	**N = 226**	**N = 33**	0.357
n events (%)	19 (23.2%)	0 (0.0%)	69 (30.5%)	8 (24.2%)	
KM median (95% CI)	80.5 (47.5–NA)	NR	64.3 (45.4–NA)	NR	
Probability at time, % (95% CI):					
12 months	86.4 (78.4–95.1)	100.0 (100.0-100.0)	88.3 (84.0–92.8)	89.2 (78.3–100.0)	
24 months	74.4 (63.1–87.8)	100.0 (100.0-100.0)	76.5 (70.5–83.0)	73.9 (57.6–94.9)	
36 months	74.4 (63.1–87.8)	100.0 (100.0-100.0)	63.9 (56.6–72.1)	59.1 (39.7–88.1)	
48 months	66.1 (49.8–87.9)	100.0 (100.0-100.0)	56.5 (48.2–66.1)	59.1 (39.7–88.1)	
60 months	66.1 (49.8–87.9)	100.0 (100.0-100.0)	52.8 (44.0–63.4)	59.1 (39.7–88.1)	

Abbreviation: N: number

ESM 10: Overall survival according to del17p/ mutation TP53					
	**VenR**	**VenR**	**BTKi**	**BTKi**	**p-value**
	**w/o del17p/TP53**	**del17p/TP53**	**w/o del17p/TP53**	**del17p/TP53**	
	**N = 68**	**N = 25**	**N = 162**	**N = 97**	0.481
n events (%)	9 (13.2%)	10 (40.0%)	49 (30.2%)	28 (28.9%)	
KM median (95% CI)	NR	80.5 (47.5–NA)	74.2 (37.9–NA)	64.3 (53.8–NA)	
Probability at time, % (95% CI):					
12 months	90.1 (82.1–98.8)	82.3 (67.9–99.8)	87.7 (82.5–93.2)	89.6 (83.4–96.3)	
24 months	76.6 (63.3–92.7)	76.4 (60.0–97.3)	70.4 (62.6–79.2)	85.2 (77.8–93.4)	
36 months	76.6 (63.3–92.7)	76.4 (60.0–97.3)	61.8 (53.1–72.0)	66.9 (56.4–79.4)	
48 months	76.6 (63.3–92.7)	66.9 (46.8–95.5)	53.3 (43.1–65.9)	62.7 (51.7–76.1)	
60 months	76.6 (63.3–92.7)	66.9 (46.8–95.5)	53.3 (43.1–65.9)	55.4 (42.7–71.7)	

Abbreviation: del: deletion, w/o: without, N: number

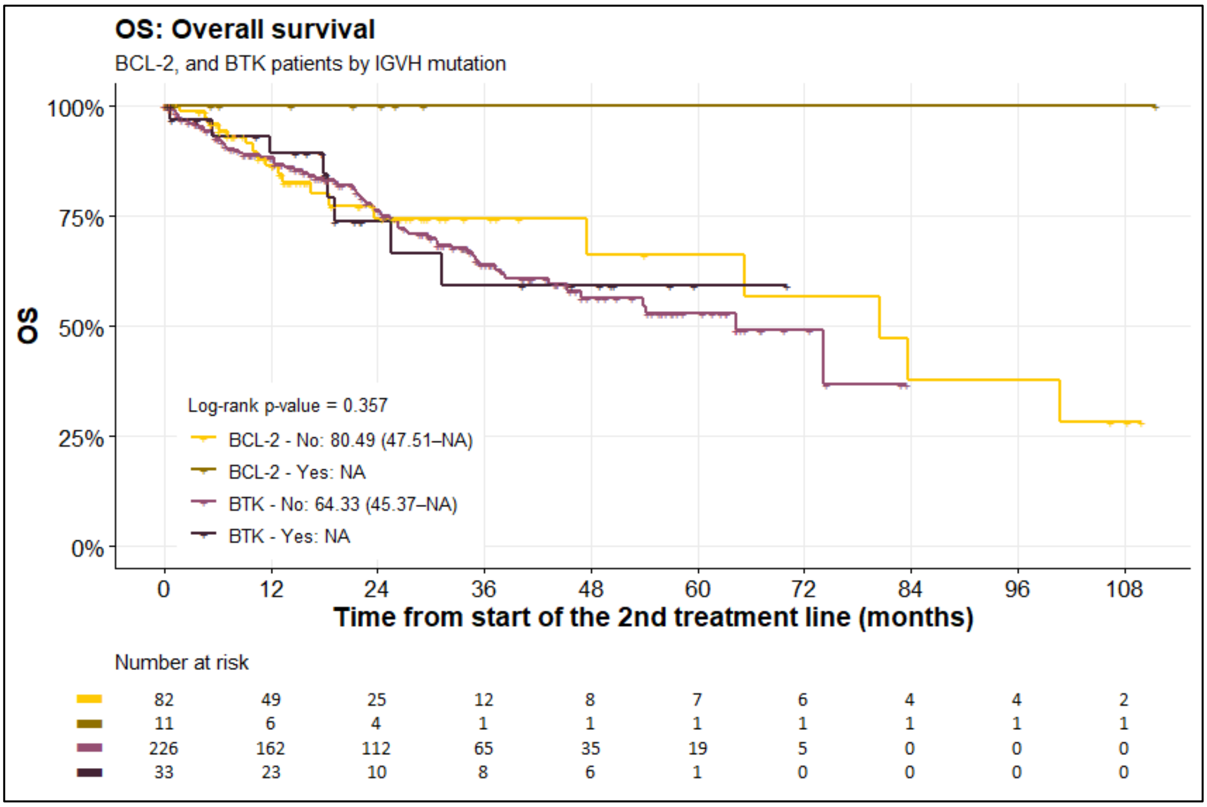

ESM 11: Overall survival according to IGHV mutation status

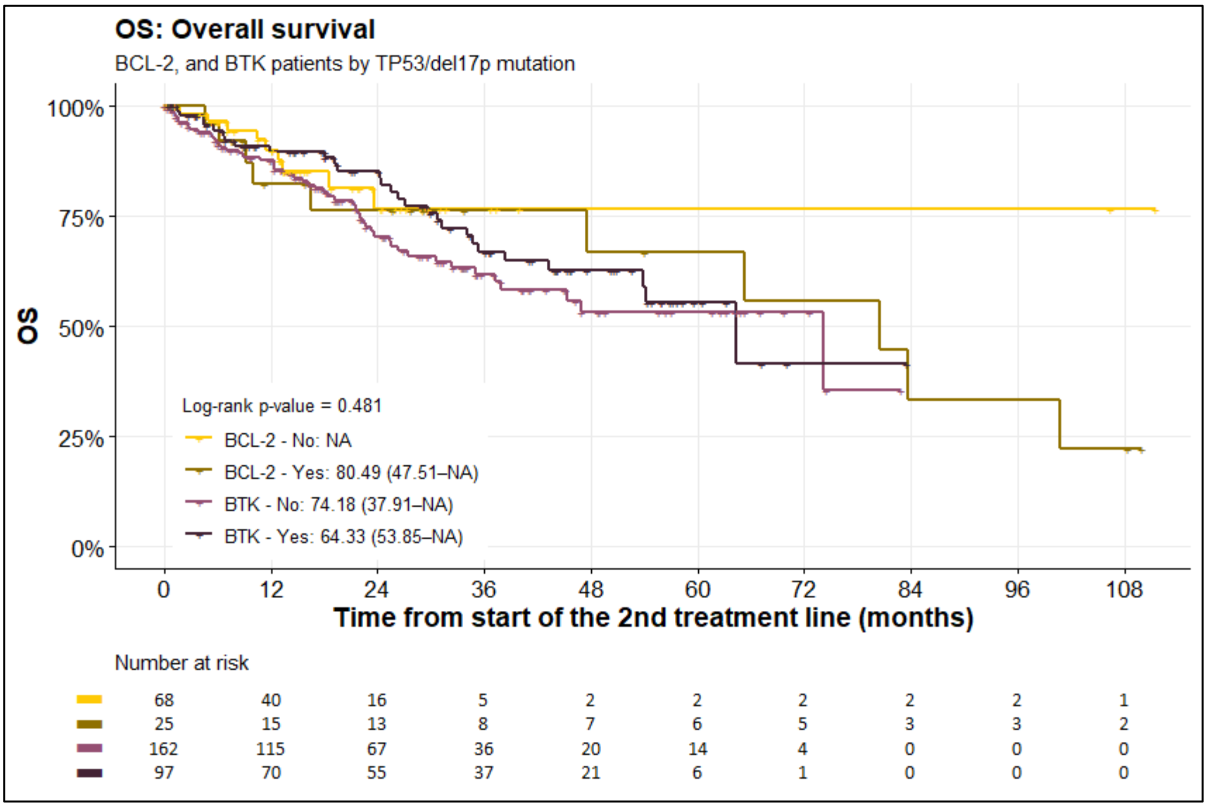

ESM 12: Overall survival according to del17p/ mutation TP53

**ESM 13: Time to next treatment**			
	**VenR**	**BTKi**	**p-value**
	**N = 85**	**N = 251**	0.764
n events (%)	14 (16.5%)	59 (23.5%)	
KM median (95% CI)	45.4 (35.3–NA)	53.4 (44.8–NA)	
Probability at time, % (95% CI):			
12 months	93.2 (87.6–99.2)	88.4 (84.1–92.9)	
24 months	90.0 (82.0–98.7)	80.7 (75.1–86.7)	
36 months	67.2 (47.3–95.4)	71.0 (63.8–79.1)	
48 months	48.0 (26.7–86.2)	56.3 (46.6–67.9)	
60 months	28.8 (11.4–72.6)	49.1 (38.5–62.6)	
Abbreviation: N: number			

**ESM 14: Time to next treatment according to IGHV mutation status**					
	**VenR**	**VenR**	**BTKi**	**BTKi**	**p-value**
	**Unmutated IGHV**	**Mutated IGHV**	**Unmutated IGHV**	**Mutated IGHV**	
	**N = 75**	**N = 10**	**N = 220**	**N = 31**	0.197
n events (%)	14 (18.7%)	0 (0.0%)	57 (25.9%)	2 (6.5%)	
KM median (95% CI)	45.4 (35.3–NA)	–	51.6 (44.6–NA)	–	
Probability at time, % (95% CI):					
12 months	92.5 (86.4–99.1)	100.0 (100.0–100.0)	86.8 (82.0–91.9)	100.0 (100.0–100.0)	
24 months	88.8 (79.9–98.7)	100.0 (100.0–100.0)	79.1 (73.0–85.6)	90.9 (75.4–100.0)	
36 months	66.0 (46.1–94.4)	100.0 (100.0–100.0)	68.7 (61.0–77.4)	90.9 (75.4–100.0)	
48 months	47.1 (26.1–85.0)	100.0 (100.0–100.0)	54.2 (44.1–66.5)	75.8 (50.6–100.0)	
60 months	45.7 (11.2–71.5)	100.0 (100.0–100.0)	46.5 (35.6–60.8)	75.8 (50.6–100.0)	
Abbreviation: N: number					

**ESM 15: Time to next treatment according to del17p and/ or TP53 mutation**					
	**VenR**	**VenR**	**BTKi**	**BTKi**	**p-value**
	w/o del17p/TP53	del17p/TP53	w/o del17p/TP53	del17p/TP53	
	**N = 61**	**N = 24**	**N = 158**	**N = 93**	**0.197**
n events (%)	6 (9.8%)	8 (33.3%)	30 (19.0%)	29 (31.2%)	
KM median (95% CI)	33.8 (29.1–NA)	53.6 (37.9–NA)	60.3 (48.1–NA)	44.8 (40.4–NA)	
Probability at time, % (95% CI):					
months	94.0 (87.6–100.0)	91.7 (81.3–100.0)	91.2 (86.3–96.3)	83.9 (76.2–92.4)	
24 months	88.2 (76.3–100.0)	91.7 (81.3–100.0)	82.2 (75.0–90.1)	77.9 (69.0–87.9)	
36 months	35.3 (8.2–100.0)	80.2 (60.1–100.0)	73.4 (64.1–84.0)	67.2 (56.2–80.3)	
48 months	35.3 (8.2–100.0)	57.3 (33.0–99.3)	62.7 (50.1–78.6)	49.3 (36.3–67.0)	
60 months	35.3 (8.2–100.0)	34.4 (13.9–84.8)	52.1 (37.0–73.5)	45.2 (31.9–64.2)	
Abbreviation: del: deletion, w/o: without, N: number					

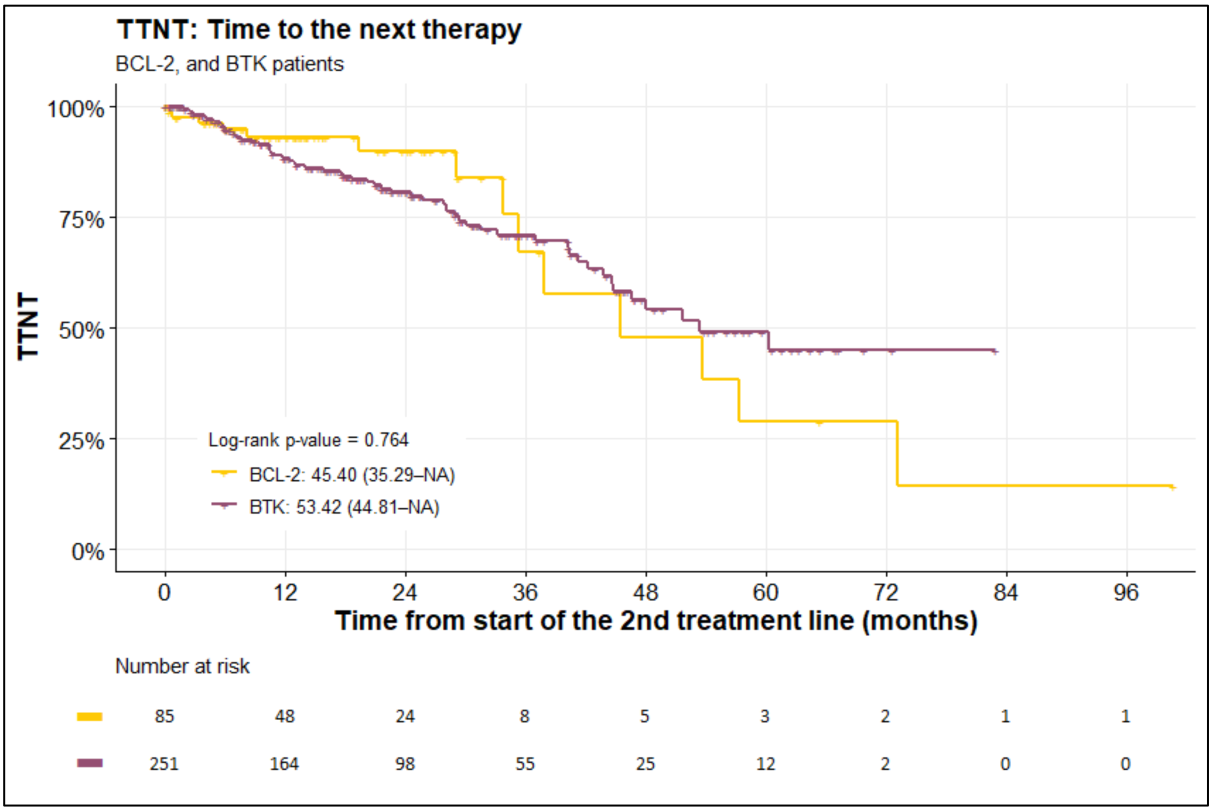

ESM 16: Time to next treatment

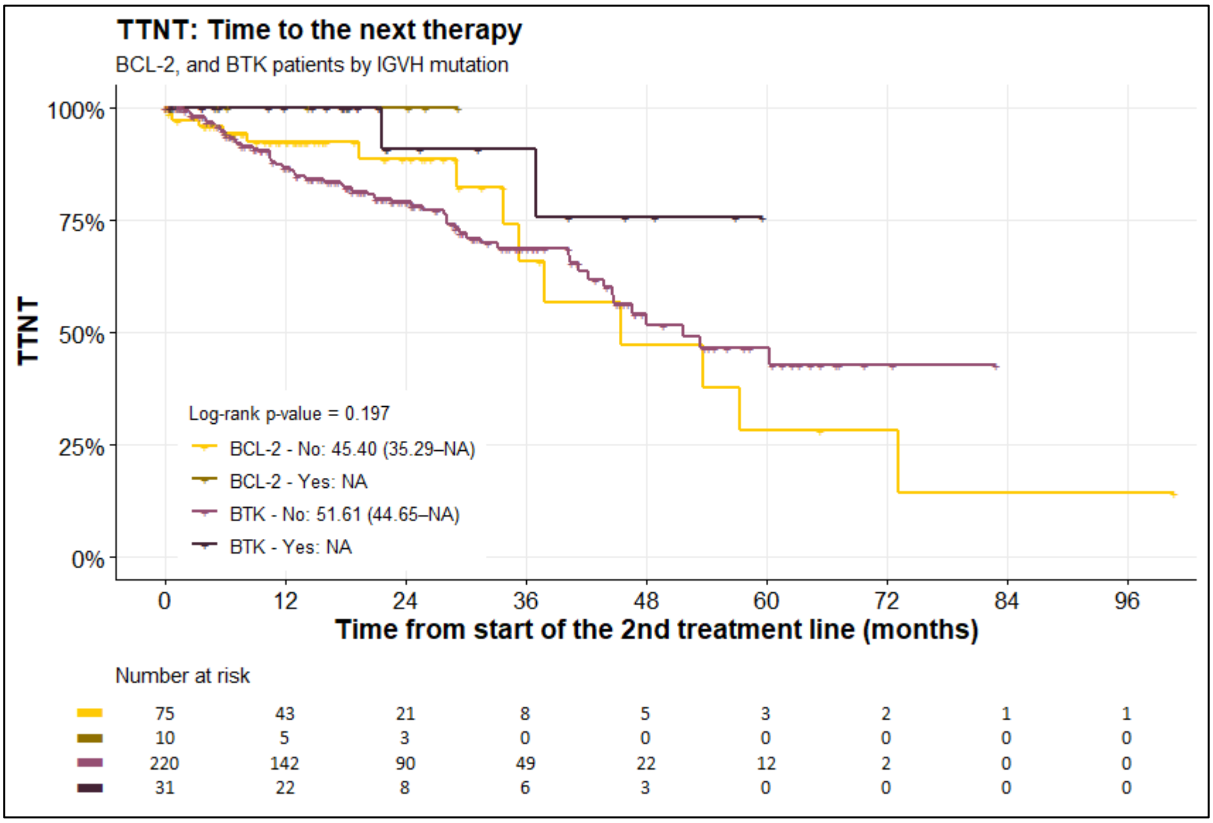

ESM 17: Time to next treatment according to IGHV mutation status

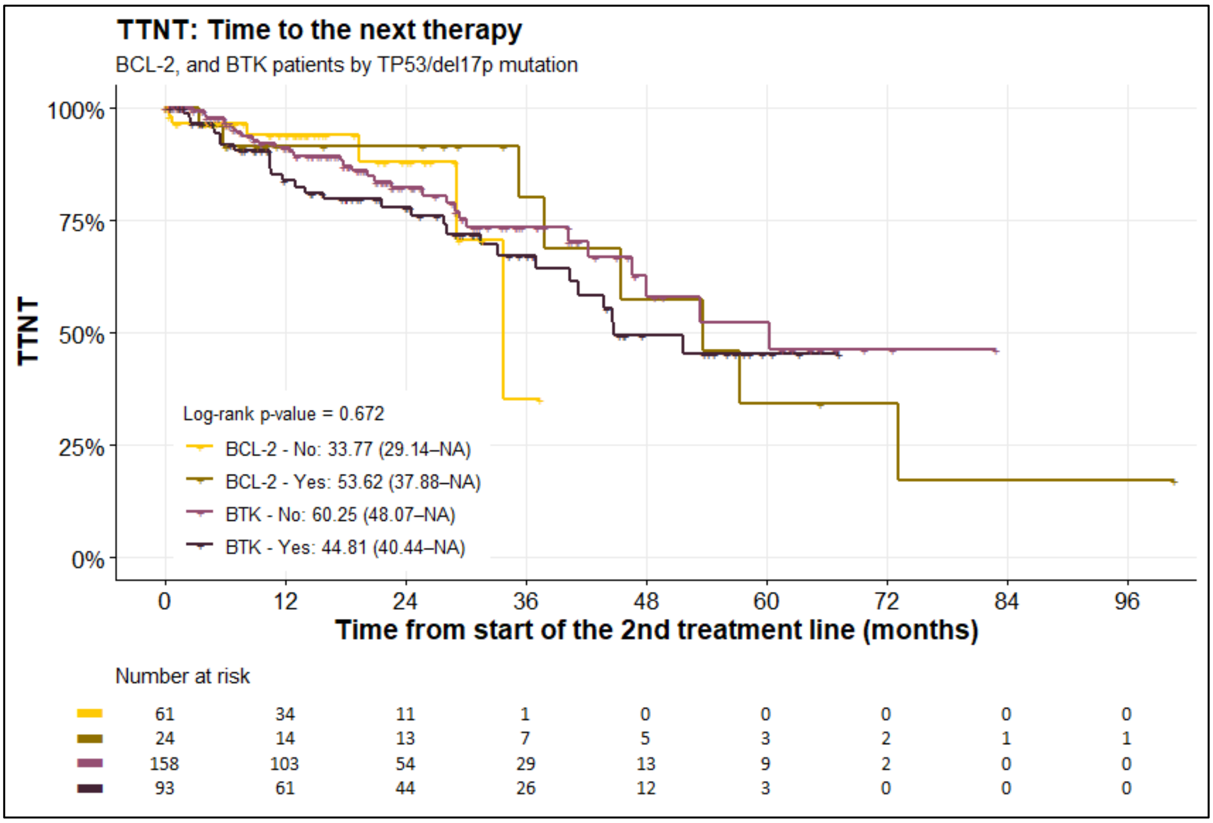

ESM 18: Time to next treatment according to del17p and/ or TP53 mutation

ESM 19: Adverse events			
	**VenR**	**BTKi**	**p-value**
**Haematologic toxicity, n (%)**	**N = 93**	**N = 259**	**<0.001**
Yes	54 (58.1%)	83 (32.0%)	
Anaemia, n (%)			**0.040**
Grade 0 - II	87 (93.5%)	254 (98.1%)	
Grade III	3 (3.2%)	4 (1.5%)	
Grade IV	3 (3.2%)	1 (0.4%)	
Neutropenia, n (%)			**<0.001**
Grade 0 - II	60 (64.5%)	234 (90.3%)	
Grade III	17 (18.3%)	12 (4.6%)	
Grade IV	16 (17.2%)	13 (5.0%)	
Thrombocytopenia, n (%)			**0.041**
Grade 0 - II	83 (89.2%)	249 (96.1%)	
Grade III	7 (7.5%)	6 (2.3%)	
Grade IV	3 (3.2%)	4 (1.5%)	
**Non-haematologic toxicity, n (%)**	**N = 93**	**N = 259**	**<0.001**
Grade I - II	7 (7.5%)	73 (28.2%)	
Grade III	6 (6.5%)	36 (13.9%)	
Grade IV	3 (3.2%)	7 (2.7%)	
Grade V	1 (1.1%)	10 (3.9%)	
Infection grade ≥3	7 (7.5%)	37 (14.3%)	0.991

Abbreviation: N/n: number

ESM 20: Other non-haematologic toxicity			
	**VenR**	**BTKi**	**p-value**
	**N = 93**	**N = 259**	
**Type of non-haematologic toxicity, n (%)**			0.076
No non-haematological toxicity	77 (82.8%)	169 (65.3%)	
Other	9 (9.7%)	15 (5.7%)	
Upper respiratory tract infections	0 (0.0%)	14 (5.4%)	
Pneumonia	2 (2.2%)	11 (4.2%)	
Diarrhoea	1 (1.1%)	5 (1.9%)	
Pyrexia	1 (1.1%)	3 (1.2%)	
Exanthema	1 (1.1%)	3 (1.2%)	
Headache	0 (0.0%)	2 (0.8%)	
Nausea	1 (1.1%)	0 (0.0%)	
Cough	0 (0.0%)	1 (0.4%)	
Vomiting	1 (1.1%)	0 (0.0%)	
Peripheral edema	0 (0.0%)	1 (0.4%)	
Abdominal pain	0 (0.0%)	1 (0.4%)	
Dizziness	0 (0.0%)	2 (0.8%)	
Arterial hypertension	0 (0.0%)	31 (11.9%)	

Abbreviation: N/n: number
